# Bioenergy Generation and Phenol Degradation through Microbial Fuel Cells Energized by Domestic Organic Waste

**DOI:** 10.3390/molecules28114349

**Published:** 2023-05-25

**Authors:** Asim Ali Yaqoob, Nabil Al-Zaqri, Muhammad Alamzeb, Fida Hussain, Sang-Eun Oh, Khalid Umar

**Affiliations:** 1 School of Chemical Sciences, Universiti Sains Malaysia, Minden 11800, Penang, Malaysia; 2 Department of Chemistry, College of Science, King Saud University, P.O. Box 2455, Riyadh 11451, Saudi Arabia; nalzaqri@ksu.edu.sa; 3 Department of Chemistry, University of Kotli, Kotli 11100, Azad Jammu and Kashmir, Pakistan; 4 Research Institute for Advanced Industrial Technology, College of Science and Technology, Korea University, Sejong 30019, Republic of Korea; 5 Department of Biological Environment, Kangwon National University, Chuncheon-si 24341, Republic of Korea

**Keywords:** microbial fuel cells, phenol, energy generation, domestic waste, rotten rice, wastewater

## Abstract

Microbial fuel cells (MFCs) seem to have emerged in recent years to degrade the organic pollutants from wastewater. The current research also focused on phenol biodegradation using MFCs. According to the US Environmental Protection Agency (EPA), phenol is a priority pollutant to remediate due to its potential adverse effects on human health. At the same time, the present study focused on the weakness of MFCs, which is the low generation of electrons due to the organic substrate. The present study used rotten rice as an organic substrate to empower the MFC’s functional capacity to degrade the phenol while simultaneously generating bioenergy. In 19 days of operation, the phenol degradation efficiency was 70% at a current density of 17.10 mA/m^2^ and a voltage of 199 mV. The electrochemical analysis showed that the internal resistance was 312.58 Ω and the maximum specific capacitance value was 0.00020 F/g on day 30, which demonstrated mature biofilm production and its stability throughout the operation. The biofilm study and bacterial identification process revealed that the presence of conductive pili species (*Bacillus* genus) are the most dominant on the anode electrode. However, the present study also explained well the oxidation mechanism of rotten rice with phenol degradation. The most critical challenges for future recommendations are also enclosed in a separate section for the research community with concluding remarks.

## 1. Introduction

Due to the fast growth of industrialization, population, and urbanization, there is a growing worry over worldwide water pollution. Providing a clean and secure water supply for people all around the globe is one of today’s most pressing environmental challenges [1]. Figure S1 demonstrates the different sources of water pollution, which is becoming a serious problem for environmental sustainability. Even at low concentrations, the presence of organic pollutants poses a threat to living organisms. Hazardous organic pollutants from groundwater and wastewater continue to require the extension of proficient and cost-effective methods [2]. Traditional water treatment technologies, such as air stripping, adsorption, oxidation, biotreatment, and chlorine action, have a number of drawbacks [3]. Industrial wastewater contains various organic pollutants such as hydroquinone, benzene, phenol, and others that must be remedied [4]. The organic pollutant that has been focused on in this investigation is phenol. The presence of phenol in water gives recipient bodies a carbolic stench and can be hazardous to aquatic flora and wildlife. Phenolic compounds are prevalent contaminants in aqueous streams due to their ubiquitous use, and they are designated priority pollutants since they are detrimental to organisms even in low quantities [5]. Due to its potential damage to human health, phenol is also a priority pollutant on the US Environmental Protection Agency’s (EPA) list of contaminants to avoid, which requires phenol levels in wastewater and drinking water to be less than 1 mg/L and less than 0.002 mg/L, respectively [6].

Microbial fuel cells (MFCs) can convert low-strength effluent and lignocellulosic material into electricity. MFCs have the ability to produce an electric current by using a broad variety of soluble or dissolved complex organic wastes in addition to renewable biomass [7,8]. Microorganisms interact with electrodes in an MFC by exchanging electrons, which are either withdrawn or supplied via an electrical circuit. MFCs are the most common form of bio-electrochemical systems, which use the metabolic activity of microorganisms to convert biomass into energy [9,10,11]. MFCs are the most sustainable, effective, and safe method of producing bioenergy [12,13]. Electrons and protons are produced when an organic substrate is oxidized, which leads to the generation of electrons. In past research, a variety of organic wastes, including domicile wastewater, waste from municipalities, and sewage sludge, were used [14,15,16]. As a source of fuel in the anode chamber, such carbohydrates are often used. In recent times, there has been a lot of focus placed on the generation of sustainable energy and the biodegradation of pollutants via the use of MFCs from food waste. This is because of the massive amounts of wasted food [17]. Numerous studies have shown that it is possible to use food waste as the organic substrate in MFCs in order to generate power [18,19,20,21]. Rice is the main meal in Malaysia, and it is often eaten twice or sometimes three times a day. In 2018, the Solid Waste Management and Public Cleaning Corporation (SWCorp) released a report stating that the highest percentage of food that is wasted daily is rotten rice, which is generated by households. This percentage increases by more than 20% during festival seasons [22]. Malaysia produces enough rice to serve 12 million people three times a day; therefore, a lot of rice waste is collected every day [22,23]. Since a large portion of today’s rotten rice is disposed of using traditional methods such as landfilling, composting, and incineration, which may contaminate groundwater, produce poisonous gases, and cause odour issues, this concern has led to serious ecological problems and health threats [24]. Conventional methods are both ineffective and impractical since they cannot fully or effectively use the energy sources from waste, such as rotting rice, that are desired. Therefore, from the standpoint of bioenergy recovery, using the rotten rice as a substrate in MFCs may be the ideal method for treating solid waste. As far as we are aware, MFCs have used food waste as a substrate of organic matter for the growth of bacterial populations; nevertheless, using phenol is a significant advancement. In this research, the focus is on how MFCs work on the degradation of phenol in the presence of rotten rice as anodic fuel. MFCs have been used to produce electricity from rotten rice as a substrate while at the same time accomplishing biological wastewater treatment (biodegradation of organic pollutant phenol). This organic pollutant also serves as a carbon source during degradation, indicating that energy production will be higher than expected.

## 2. Results and Discussion

### 2.1. Bioenergy Generation and Polarization Trend

**Figure 1:**
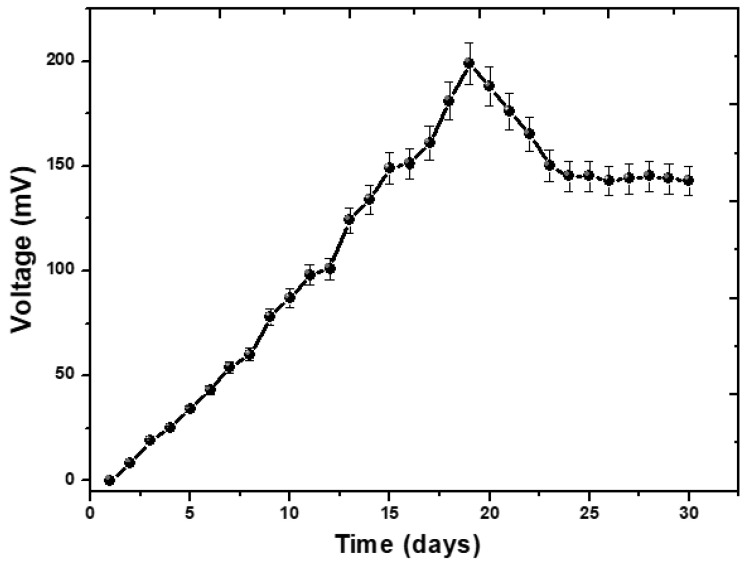
The voltage potential performance of phenol by MFCs.

The complete process of operating the MFCs with the aim of generating bioenergy took place over the course of one month. At the beginning of its operation, the MFCs’ reactor was loaded with an external resistance of 1000 Ω. During the process of phenol degradation, an increase in the cell’s potential (measured in mV) and current (measured in mA) were noted. As the experiment turned on, the voltage in the reactor increased as a result of the proliferation of bacteria that sprang from the rotten rice. It is possible that the addition of rotting rice may increase the demand for carbon and oxygen, which will be explored in a further study in connection with the performance of MFCs. The formation of biofilm on the surface of the anode has an effect on the amount of voltage that is produced. The formation of the biofilm, which was necessary in order to transport electrons to the anode, took many days. On day 19, a voltage that was significantly high at 199 mV was recorded. The findings suggested that the voltage produced by the MFCs technology may be explained by the fact that the microorganisms were feeding on rotten rice. During the first 19 days, the voltage steadily increased from 0 mV to 199 mV before entering a drop phase, as seen in Figure 1. This phase lasted for the remainder of the study. After day 19, there is a steady trend towards a lower voltage, which is eventually followed by a voltage that is constant. This indicates that all of the organic substrate has been completely oxidized, and that the bacterial species involved are unable to produce electrons [25]. The addition of phenol, which was intended to boost the voltage, did not have the anticipated effect, and instead caused the voltage to decrease. There was no longer a movement of electrons from the anode to the cathode, and the voltage remained stable, as shown in Figure 1. This finding suggests that the bacterial species have completed their life cycle.

**Figure 2:**
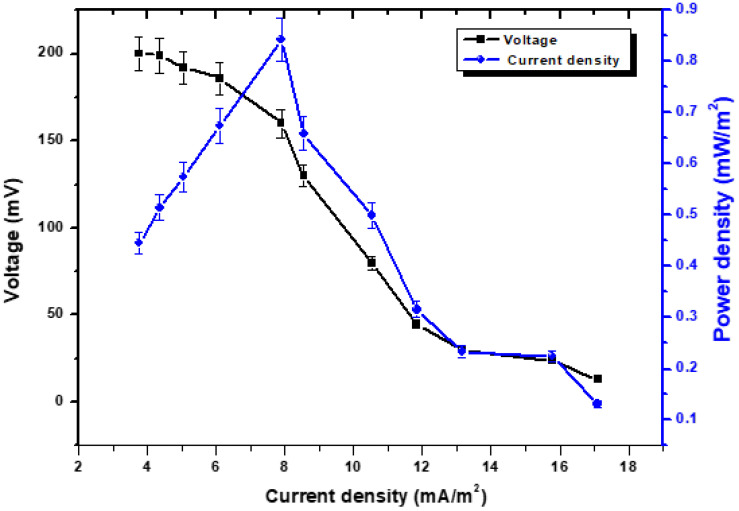
Polarization behavior with the relationship of voltage, power density vs. current density.

This illustrates how stable voltage, current density, and power density are interconnected. According to an external source with variable external resistance, the recorded voltage is measured [26]. While the current or current density is measured in accordance with Ohm’s law, the voltage is obtained by the variable resistor box [27]. As can be seen in Figure 2, the activation potential dropped from 200 mV to 13 mV while the resistance increased from 5 kΩ to 100 Ω. It is possible that this could be linked to the loss of heat energy that occurs when oxidation or reduction is initiated, or it might be due to the loss of electrons that occur from terminal proteins in bacterial cells to the anode surface [28]. The voltage dropped from 220 mV to 13 mV, while the rise in the external resistance brought it from 5 k Ω to 100 Ω. However, if the external resistance was reduced from 5 kΩ to 100 Ω, there was a significant increase in the rate at which the power density increased. This was calculated in the MFCs at an external resistance of 100 Ω, which served as the cell design point for the MFCs in the subsequent studies. The overall power density was found to be 0.85 mW/m^2^, and the current density was found to be 17.10 mA/m^2^. Although the achievable stability was not quick at reduced resistance, progress was being made towards power density. The voltage destabilization was quickly reduced, and even at low levels of external resistance, it remained fairly resilient. According to Abbas et al., [29], a strong electron discharge may be the cause of a persistent drop in potential and stability values at lower resistances. Reduced resistances allow electrons to move more rapidly across the circuit, which results in maximum current levels and power densities while having a lower degree of stability [30]. It is referred to as an ohmic over potential, and it was caused by the electrical resistance of the electrode as a result of the energy loss that occurred during the transport of protons via the cathode. Due to the enormous oxidative pressures that are present at the anode, the voltage will begin to drop as the current density continues to increase [31].

### 2.2. Conductivity Test

**Figure 3:**
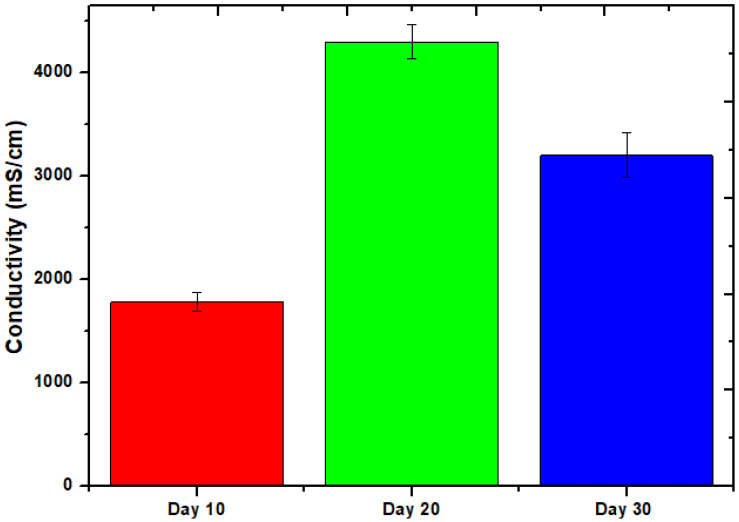
Conductivity of MFCs at different times.

Figure 3 depicts the evolution of this phenomenon over time as the conductivity of the cell changes. The conductivity measurements were collected at a variety of times during the course of the 30-day experiment. From day 10 to the very final day, the value ranged from 1780 mS/cm to 3210 mS/cm, although day 20 had a value of 4300 mS/cm. The statistics are consistent with a large amount of power being produced on day 20, given the high conductivity. The largest voltage seems to have been produced on days 19 and 20 of the experiment based on the pattern of voltage generation. The relevance of the system remains true throughout an operation basically unaltered. Long-term exposure to environmental pressures such as pH shifts, organic substrate degradation, and temperature swings causes the system’s efficiency to gradually decrease. A related study was also published recently by Rojas-Flores et al. [32] to explain the conductivity effect.

### 2.3. Cyclic Voltammetry and Electrochemical Impedance Spectroscopy Study

**Figure 4:**
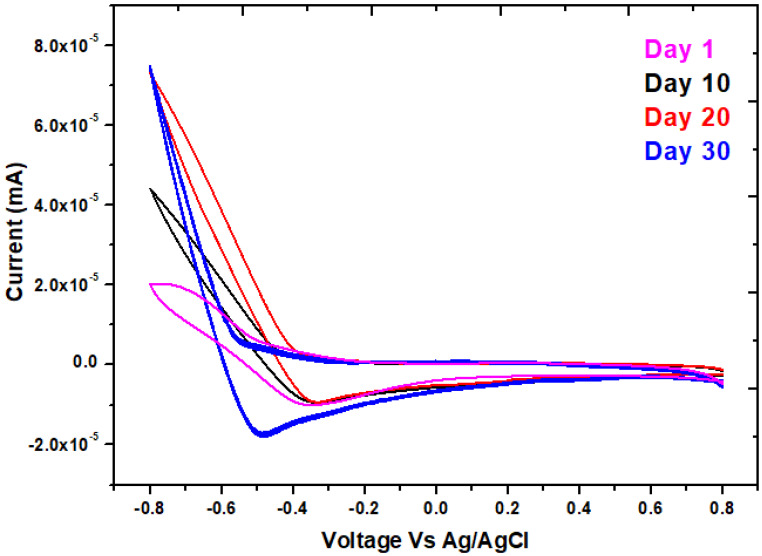
Cyclic voltammetry for the MFCs with phenol at day 1, 10, 20, and 30, respectively.

It is an electrochemical approach that interprets the system’s electrochemical behavior and is most typically used to characterize electron transfer interactions on the interface biofilm of the anode in MFCs [33,34]. In the anodic chamber of the MFCs, a forward peak was seen on day 30 at 0.8 V, showing a precise oxidizing property, whereas a reverse peak was seen at −0.5 V as shown in Figure 4. As a result, a solid redox loop in MFCs systems was attained to guarantee that active microbial communities can transfer electrons. On the 30th day, when the highest Faradaic current is present, the CV shows maximal reversible peaks exclusively owing to reduction electron-transfer. It has been suggested that the migration of an electron from a redox potential to the anode surface may be implicated in the process of controlled electron transfer [35]. Oxygen may be responsible for the highest levels of oxidation and reduction in MFCs [36]. The organic substrate in the MFCs was the main contributor to the oxidation rate. The exoelectrogenic density on the electrode grew with time, increasing the potential values. The amount of metabolites in the feedstock changes as exoelectrogenic levels grow, which affects the electrical solution’s flexibility and conductivity [37]. It was investigated how various days—the first, tenth, twentieth, and thirtieth—affected the inoculum’s electrocatalytic activity. In light of the fact that the 30th day curve has the highest peak of all the curves, its area may suggest that this day performs better than others in terms of stability and specific capacitance [38,39]. According to Table 1, this maximum peak denotes biofilm development over MFCs with the highest specific capacitance of 0.00020 F/g throughout all days of curves. The entire anode potential for MFCs grew as the anode’s biofilm developed. The bacterial cell membrane’s redox enzymes and the superficial charge storage capabilities in the bacterial cytoplasm may be responsible for this enhancement in anode capacity [40]. 

**Figure 5:**
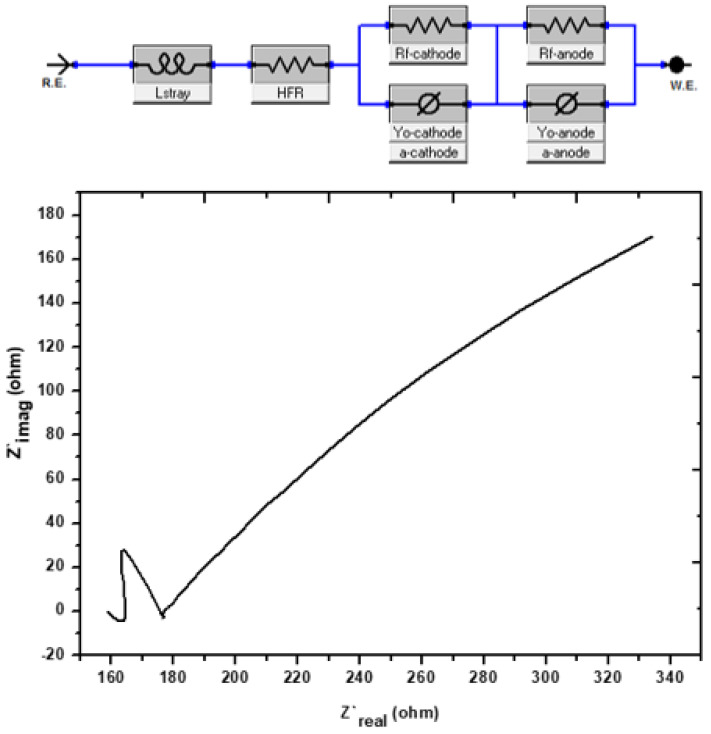
Nyquist graphs of the investigation’s impedance spectra on day 30 of the experiment.

Figure 5 presents the Nyquist plot, which was derived using the EIS technique as an effective means of demonstrating the bio-electrochemical phenomena that took place in the MFCs. The Nyquist plot usually consists of either a straight line or a semicircle. In this plot, the straight line represents the diffusion-regulated reaction, and the diameter of the semicircle represents the resistance to the charge transfer through EIS [41]. This plot is displayed in Figure 5, as it was previously described by several researchers in the same pattern [42,43,44]. On the 19th day after the growth of the biofilm, the fuel cell was put through tests with the assistance of an EIS-potentiostat. When it applies to electrical accessibility, semi-circles or semi-bent lines imply tremendous mobility, but straight lines with a high Z’_img_ (Ohm) represent limited mobility [45]. On day 19, proof was supplied indicating the biofilm had entirely established itself and was now stable. This evidence was provided in the form of enhanced electronic mobility along with semi-bent curves. The MFCs internal resistance was 312.5 Ω. Having a low charge resistivity and a low internal resistance makes it feasible for electrons to move more readily. This is because charge resistivity is low [46]. After reaching its peak on day 19, the voltage output gradually started to decline in the days that followed. When there is internal resistance, there is a corresponding reduction in the quantity of electron mobility. The electrolytes and the efficiency of the organic substrate are additional factors that have an influence on the internal resistance [47]. 

### 2.4. Oxidation of Rotten Rice

The MFCs technique is mostly dependent on the activities of the different bacterial species. In this work, researchers identified a number of bacterial species that are well-known exoelectrogens and organic pollutant-reducing species. The organic substrate in MFCs is oxidized by bacterial species, which results in the generation of bioenergy [48]. In this study, bacterial species oxidize the rotten rice waste, a polysaccharide sugar, which is first converted into simple sugar. The oxidation-reduction reaction can be written as:

Anode reaction: Rotten rice → C_6_H_12_O_6_ + 6H_2_O →6CO_2_ + 24H^+^ +24e^−^

Cathode reaction: 24H^+^ + 24e^−^ + 6O_2_ → 12H_2_O

Overall: C_6_H_12_O_6_ + 6O_2_ → 6CO_2_ + 6H_2_O + Energy + Biomass

The anode is responsible for producing electrons and protons, which are then delivered to the cathode. The proton is able to move directly from the anode to the cathode, but the electrons have to travel via an external circuit. In addition, there is a period of time during which electrons are transferred from the bacteria to the anode electrode before they are transported from the anode to the cathode. In the literature, the electron transfer mechanisms (bacteria to anode) are well explained, and they are also shown in Figure S3. 

### 2.5. Biodegradation Performance of Phenol

**Figure 6:**
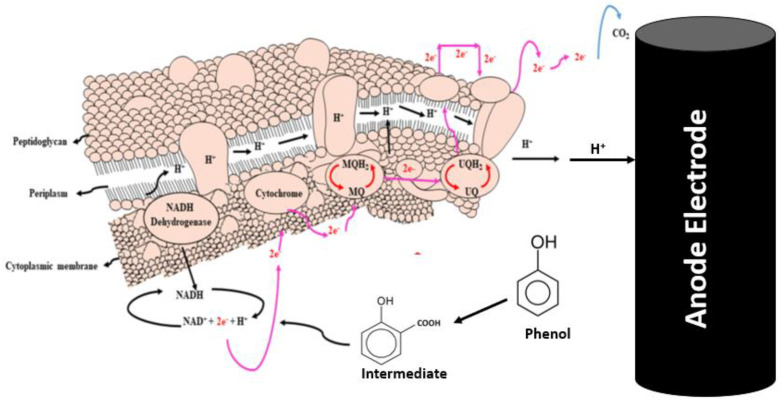
Graphic idea of phenol biodegradation through bacterial communities (modified according to present study from reference [52] with permission from Elsevier to reuse it).

**Figure 7:**
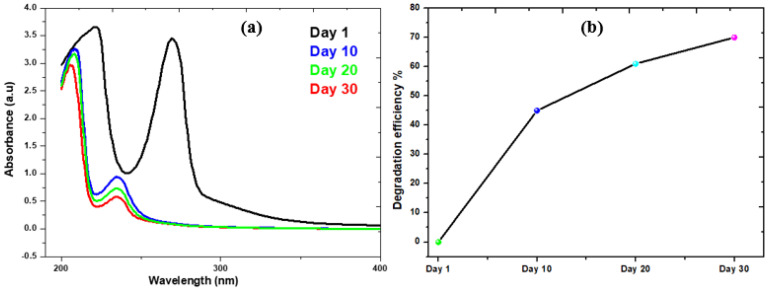
(**a**) UV-Spectra of Phenol Collected from MFCs; (**b**) Degradation percentage of phenol in this system.

The addition of rotten rice waste as an organic substrate improved the rate of biodegradation by enhancing the metabolism of bacteria. The present study achieved 70% degradation efficiency of phenol by this MFCs investigation. Two distinct microbes—bio-degradative and electroactive microbes—participated in the biodegradation of the phenol. While electroactive bacteria contribute to the biodegradation of intermediates, the bio-degradative bacteria target phenol and cause a full ring-cleavage process. Phenolic acid (intermediate) is produced via a complete carboxylation process mediated by the carboxylase phenol. However, subsequent phenol cleavage by electroactive bacteria results in the production of molecules of CO_2_ and electrons, as shown in the electrochemical processes listed below. Figure 6 illustrates the evidence in support of the degradation of phenol and phenolic acid into the final product. The absorbance peaks of phenol at different time intervals are shown in Figure 7a. On the basis of UV curves, the calculated biodegradation efficiency is shown in Figure 7b. As can be seen on curve day 1, the absorbance peak of phenol at λ_max_ = 275 nm occurs prior to the operation of the reactor and is caused by the transition π→π* of aromatic (C=C) [49]. Following the completion of the reactor cycle, the phenol peak of the curve (day 1) has moved to a maximum wavelength of 245 nm (day 10). The process of phenol’s biodegradation into phenolic acid begins at this point [50]. Phenol is transformed into phenolic acid. As a consequence of the delocalization of electrons, the intermediate has a higher degree of conjugation than phenol [51]. The biodegradation of the intermediate phenolic acid was essentially degraded, as seen by the curve on days 20 and 30, which had a band at 245 nm. The biodegradation efficiency calculation also shows a gradual degradation trend. In light of these findings, the anodic and cathodic processes that take place in the MFCs are described by the electrochemical equation given below.

Anode reaction:

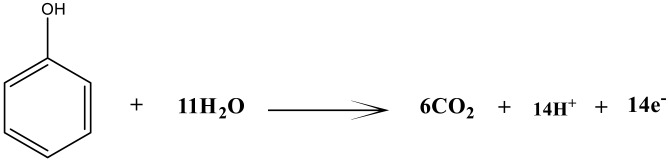



Cathode reaction:

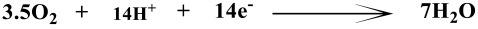



Overall electrochemical reaction:

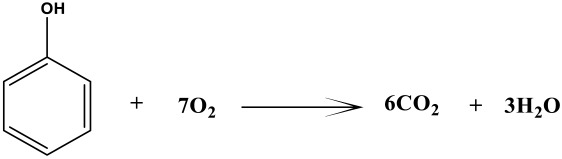



### 2.6. Biofilm Study 

**Figure 8:**
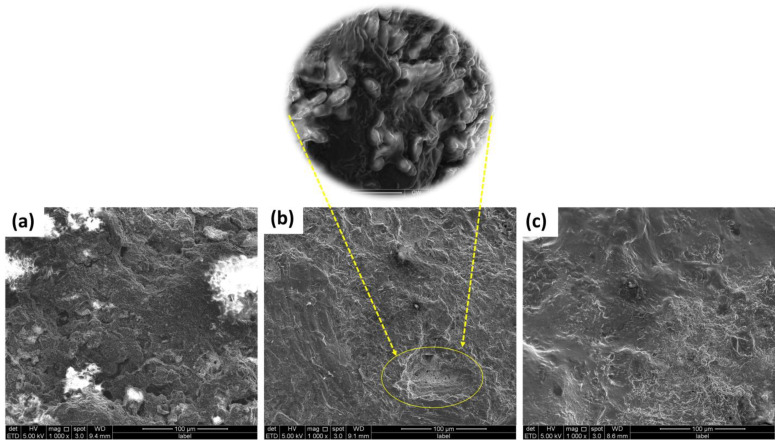
SEM images of (**a**) Graphite electrode before MFCs operation; (**b**) Graphite electrode as anode after MFCs operations; (**c**) Graphite electrode as cathode after MFCs operations.

**Figure 9:**
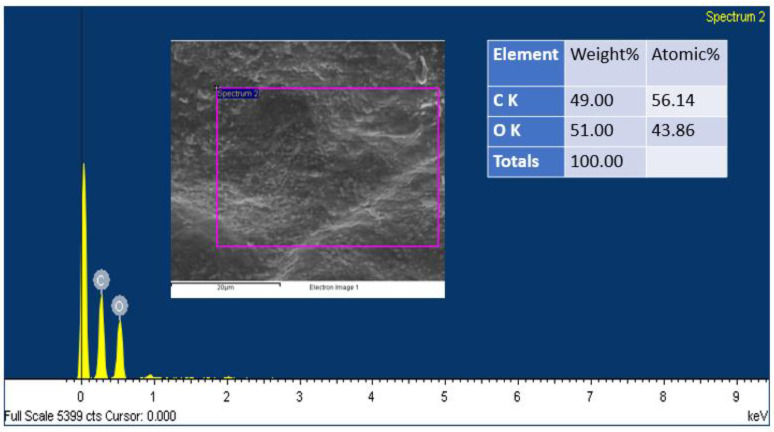
EDX spectra of anode electrode of the present study.

In addition, SEM analysis was carried out on the electrode (both the anode and the cathode) before and after the operation to analyze the bacterial morphology that was present on the surface of the electrode. A distinct distinction can be seen in the SEM of untreated graphite, treated anode, and treated cathode, as shown in Figure 8a–c. Figure 8a displays a planar morphological surface, due to the fact that it was a commercially available graphite electrode that had never been used. Meanwhile, the anode electrode had a thick filamentous structure on its surface, indicating the existence of bacterial species colonies that are most likely biodegradative and electroactive microorganisms. The cathode also had some clusters of bacteria; however, these clusters were not as dense or well developed in comparison to those on the anode electrode. On the surface of the anode electrode was a biofilm that was very thick and had developed to a mature state. This biofilm was scraped to identify the bacteria. The anode SEM had a morphology that resembled a tube or rod, and this form predominated in the pictures. It is evidence of the species of bacteria that are now known to possess conductive pili. Conductive pili are structures that are filamentous and take the form of rod-shaped wires; bacteria utilize these pili to transmit electrons from the cell to the anode electrode [53]. This is a significant and direct contact that takes place between the cell of the bacterium and the surface of the anode. There are a number of experiments that have been performed that have shown that the conductive pili are able to effectively transmit electrons from bacterial cells to the anode surface [54,55,56]. In addition, by referring to this research, it is possible to draw the conclusion that the dense development of biofilm is an indicator of the anode’s high level of biocompatibility with regard to living organisms. Due to the high sugar content of the organic substrate that is given, the limited species of bacteria that are present are located in very dense conditions on the surface of the biofilm [57]. An EDX analysis of the biofilm was carried out in order to determine whether or not certain components were present on the anode’s surface. Based on what we see in Figure 9, we are able to draw the conclusion that phenol does not have a negative impact on bacterial populations. Since the anodic biofilm solely contains carbon and oxygen at the end of operation. The results of the investigation demonstrated, on the whole, that the presence of the organic pollutant during the operation of the MFCs did not demonstrate any toxicity towards the bacterial population. 

### 2.7. Bacterial Identification 

**Figure 10:**
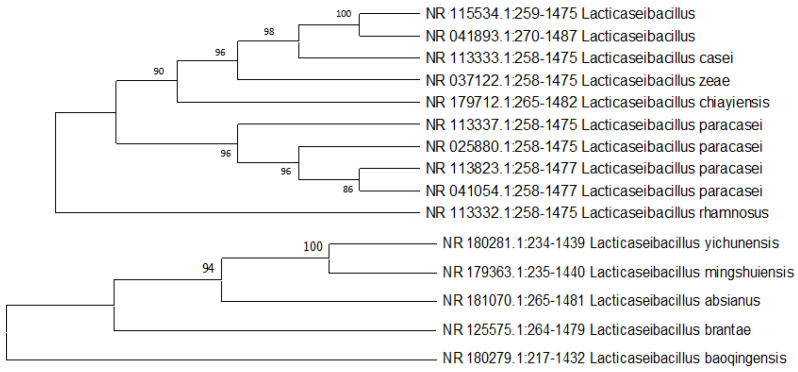
Phylogenetic study of bacterial species from anodes of the MFCs.

In the framework of biological research, a process of bacterial isolation and identification was carried out in order to determine the species of bacteria. In an effort to determine the species of bacteria, the anodic biofilm was analyzed. The list of the most prevalent identified bacterial species is shown in Figure 10. There are different kinds of bacterial species that are classified as conductive pili-type species. It indicates that the rod-shaped appendages that may be observed in the SEM pictures are providing evidence that conductive pili-typed species exist. *Bacillus* is, in general, the most prevalent genus on the biofilm’s surface. The detected bacteria are genus members that have previously been shown to be biodegradative and electroactive in a variety of earlier investigations and published works of literature. In addition, Nimje et al. [58] reported that the power density of the *Bacillus* strains when used as a biocatalyst was 0.000105 mW/m^2^. However, a few investigations demonstrated that the found bacterial species (Figure 10) are well known as biodegradative and electroactive species, which we also found in our experiment [59,60]. 

## 3. Experimentation

### 3.1. Chemical and Materials

The wastewater was collected from a pond located at Bayan Lepas, Penang, Malaysia (6.3195° N, 100.1132° E). The rotten rice waste was acquired from the Café at the Universiti Sains Malaysia (USM). Materials were acquired from a variety of sources, such as commercial graphite rods (FUDA 2B Lead; New York, NY, USA), distilled water, and phenol (Sigma-Aldrich, St. Louis, MO, USA).

### 3.2. Inoculation of MFCs

The pond effluent collected from a local pond (Bayan Lepas, Penang, Malaysia) was treated with 500 mg/L of phenol to prepare an inoculation of MFCs. In this study, as an organic substrate, 750 g of rotten rice waste was used. Synthetic wastewater was used to refer to the wastewater that included organic pollutants. Several pond effluent and synthetic wastewater physicochemical properties are listed in Table 2. We used a pH meter (EUTECH instrument-700, New York, NY, USA), a thermometer (GH, ZEAL LTD, London, UK), and an electrical conductivity meter (ESEL, Ambala, India) to measure the conductivity, pH, and temperature, respectively. 

### 3.3. MFCs Set Up and Operational Protocol

In this experiment, a single-chamber microbial fuel cell (MFC) was developed to produce recycled water from wastewater and produce energy. For the degradation phenol, the MFC measured 16.5 cm and 13.5 cm in height and diameter, respectively. The total operational capacity of the MFC was 1000 mL; however, only 100 mL of phenol stock solution and 1000 mL of sewage were used to pollute 750 g of rotten rice. The MFC had a total of 1000 mL of wastewater. After that, commercial graphite rods measuring 11.5 cm × 1 cm (h × r) for the anode and the same length for the cathode were vertically put into the MFC in order to create the cathode and anode, respectively. There was a distance of 17 cm between the anode and the cathode. By adding a 1000 Ω external resistance, the electrodes were linked with copper wire. Experiments using rotten rice were carried out using MFCs for a period of one month at room temperature. Figure S2 depicts the MFC reactor used in this research.

### 3.4. Electrochemical Measurements and Calculations

The potential voltage between the anode and cathode was measured using a digital multimeter (UNI-T UT33A, China) once each day, and the resulting current was calculated using Ohm’s law. Using Equations (1)–(4) below, we were able to determine the current density (CD), power density (PD), and internal resistance (r). The voltage unit is ‘mV’, CD has mA/m^2^, and PD has mW/m^2^:

Voltage Output (V) = IR(1)

(2) $$\mathrm{Power}\;\mathrm{Density}=\frac{V^{2}}{\mathrm{RA}}$$

(3) $$\mathrm{Current}\;\mathrm{Density}=\frac{I}{A}$$

(4) $$\mathrm{Internal}\;\mathrm{Resistance}=\left[\frac{E-V}{V}\right]R$$

where “A” represents the anode electrode surface area; the “I” represents the current; the “R” represents the internal resistance; and “E” represents the electromotive force. Measurement of the electromotive force was carried out with the help of an open circuit voltage (OCV). The slope of the polarization curve was used in conjunction with a variable resistance box that had a range from 5000 to 100 Ω in order to determine the MFCs’ internal resistance. Further, conductivity tests are also carried out every 10 days by using the conductometer.

#### 3.4.1. Cyclic Voltammetry (CV) 

The cyclic voltammetry (CV) technique was used to investigate the redox reactions occurring on the electrode surface. Using a scanning rate of 10 mV/s and a potential range of +0.8 V to 0.8 V, the electrode surface was measured at the 1st, 10th, 20th, and 30th day intervals, respectively. The platinum wire and the anode each took their place as the counter and working electrodes, while the Ag/AgCl mixture was used as the reference electrode. The reference electrode was found by using the potential of the electrodes. The particular capacitance, denoted by the notation Cp (unit is F/g), was defined as the sum of the data from the cathode and the anode when expressed as a fraction of a gramme. Cp was computed from CV data using the following Equation (5), where A represents the area of the CV curve, m represents the loaded sample amount in the CV instrument, k represents the CV scan rate in mV/s, and (V2 − V1) specifies the potential range of the CV:

(5) $$C_{P}=\frac{A}{2\mathrm{mk}\;\left(V_{2}-V_{1}\right)}$$

#### 3.4.2. Electrochemical Impedance Spectroscopy (EIS)

Electrochemical Impedance Spectroscopy (also known as EIS) was used to study the influence of the anode’s resistance on voltage at many points in time during the experiment. On day 19, EIS research was carried out using MFCs in their operational mode, with the frequency range being from 100,000 Hz all the way down to 0.1 Hz. The amplitude of the alternating current (AC) was set to around 1 mV so as to prevent the adhesion of biofilm and to reduce the amount of disturbance caused to the steady-state system.

### 3.5. Biological Characterization

#### 3.5.1. Biodegradation of Organic Pollutant

To assess biodegradation efficiency, phenol contents were measured under an ultraviolet–visible (UV-Vis) light source using a Shimadzu UV-Vis 2600 Spectrophotometer. Every 10 days, a sample of around 1 mL from the phenol MFCs was obtained, diluted to 50 ppm, and the organic pollutant level was calculated. Calculating the biodegradation efficiency % required using Equation (6), where T_o_ was denoted as transmittance of the standard, T as transmittance of water sample, and [T_o_] as concentration of the standard solution.

(6) $$\mathrm{Biodegradation}\;\mathrm{efficiency}\;\%=\frac{T_{o}-T}{\left[T_{o}\right]}\times 100$$

#### 3.5.2. Bacterial Identification and Biofilm Studies

The purpose of this experiment was to ascertain the species of bacteria that are present in MFCs by isolating and identifying the bacteria. Obtaining pure bacterial cultures may be accomplished via the techniques of serial dilution and plating bacteria through the use of the streaking method. A material may be diluted into a solution using a method known as serial dilution. After the material is diluted, it is spread out on nutrient agar and left to rest for three days. The method of streak plate may be used to separate bacteria from a mixed population so that they can then be cultivated in a pure culture. The inoculum was spread throughout the agar surface in this manner, which resulted in the bacteria population becoming less dense. The polymerase chain reaction (PCR) technique was used in the process of creating bacterial genes. In order to amplify the genes, both a forward primer and a reverse primer were used. The cloning of the PCR-generated result was accomplished with the help of a cloning kit manufactured by Invitrogen and located in Carlsbad, CA, USA. After having their DNA sequenced, bacterial strains were uploaded to the GenBank database. Scanning electron microscopy (SEM) was used in order to investigate the development as well as the stability of the biofilm and the surface appearance. After going through the process of having the organic pollutant content on its surface reduced by synthetic wastewater, the anode in the SEM images shows signs of bacterial growth on its surface. At the conclusion of the procedure, an energy dispersive X-ray, abbreviated EDX, was taken from the biofilm-anode electrode in order to study the impact that the hazardous organic pollutant had on the biofilm. 

## 4. Challenges and Future Perspectives

MFC technologies are environmentally viable ways to produce electricity and degrade organic contaminants from wastewater, according to modern research. MFC technologies have certain major drawbacks, including design, cost, and performance. Thus, they have never competed in renewable energy or wastewater treatment. MFCs can produce enough net energy to replace energy from organic material oxidation using waste and inorganic carbon under specific circumstances. MFC systems can biologically convert chemical energy into electrical form, enabling them to handle a broad variety of chemical substrates at varying concentrations. MFC technology helps researchers investigate electrochemical, biochemical, microbial, and material surface reactions under controlled circumstances [61]. They research how materials, chemical compounds, and feedstock substrates impact them. This approach helps us grasp MFCs’ larger-scale difficulties. In order for MFC technology to be sustainable on a commercial scale, steps need to be taken to reduce the high operating costs and improve the technique’s power generation. MFCs have a high degree of capital cost because they need costly electrode materials, such as catalyst, current collector, and separator materials. This contributes to the overall cost of the device [62]. Recently, attention has been paid to this issue by a number of scholars. The use of an electrode that is produced from biomass is one potential approach to resolving the cost concerns surrounding electrodes. In comparison to the literature, the findings that were obtained by a research team that concentrated on graphene-derived electrodes that were synthesized from waste biomass were much more favorable [63,64,65]. As a result, several types of biomasses and their most recent modifications might lead to an answer to this issue. There are several operating issues, such as pH, temperature control, potential range, etc., but several scholars optimized it at different ranges and mostly agreed to operate the MFCs at natural conditions, such as pH 7 at room temperature. They also believed that they could minimize the internal resistance by improving the MFCs design and reducing the distance between the anode and cathode [66]. In spite of these relatively minor concerns, the most important problems involve electron transportation and generation. The migration of electrons is closely connected to the development of electrodes, as mentioned above. The oxidation of the organic substrate determines whether or not it is favorable for bacterial species to oxidized substrate quickly, which is a prerequisite for electron production. One of the potential solutions is using material that has been generated from waste as an organic substrate [67,68,69]. Finally, integrating MFCs with other wastewater treatment methods could boost the efficacy of treatment and, as a result, significantly lower total power consumption [61].

## 5. Conclusions

The dual-purpose use of MFCs in environmentally friendly wastewater treatment is gaining in popularity. It leads to a more efficient degradation of organic compounds such as phenol and produces bioelectricity that can be channeled and ramped up to meet rising energy needs. In this work, we used MFCs driven by rotting rice to effectively generate a significant power density and efficiently biodegrade phenol. The optimization of variables such as internal resistance, polarization, specific capacitance, and voltage production is well discussed. As a consequence of the bacterial species adhering to the anode surface, a denser and more uniform biofilm was formed, according to SEM data. Bacterial species have developed the capacity to produce bioelectricity. The biofilm research and bacterial species identification methods showed that phenol is successfully degraded while producing energy with no negative side effects. According to all evidence, the present study followed the electron transfer mechanism (the mechanism is well explained in the previous literature) via conductive pili. 

## Figures and Tables

**Table 1:** The values of Cp at several specific times.

Measurement Days	Capacitance (F/g)
1st	0.00002
10th	0.00014
20th	0.00018
30th	0.00020

**Table 2:** Characteristics of pond effluent and synthetic wastewater.

Parameters	Pond Effluent	Synthetic Wastewater
Color	Cloudy	Cloudy
Electrical conductivity	15.40 µs/cm	24.10 µs/cm
pH	6.99	5.45
Temperature	24 °C	24 °C
Odor	Bad	Bad
Phenol	0 mg/L	500 mg/L

## Data Availability

All the data are included in the text.

## Supplementary Materials

The following supporting information can be downloaded at: https://www.mdpi.com/article/10.3390/molecules28114349/s1, Figure S1: Different sources of water pollution (Adapted from: https://www.filterwater.com/t-articles.water-pollution.aspx, accessed on 8 May 2023); Figure S2: MFCs Set-up for phenol degradation; Figure S3: Different mechanisms of the electron transfer from bacterial species to anode electrode in MFCs (Adapted from reference [70] with Elsevier permission). References [24,66,70,71,72] are cited in Supplementary Materials.

## References

[1] Guerrero–Barajas C., Ahmad A., Ibrahim M.N.M., Alshammari M.B., Shahid-ul-Islam, Shalla A.H., Shahadat M. (2023). Advanced Technologies for Wastewater Treatment. Green Chemistry for Sustainable Water Purification.

[2] Tijani J.O., Fatoba O.O., Madzivire G., Petrik L.F. (2014). A review of combined advanced oxidation technologies for the removal of organic pollutants from water. Water Air Soil Pollut..

[3] Parveen T., Umar K., Mohamad Ibrahim M.N. (2020). Role of nanomaterials in the treatment of wastewater: A review. Water.

[4] Idris M.O., Kim H.-C. (2022). Exploring the effectiveness of microbial fuel cell for the degradation of organic pollutants coupled with bio-energy generation. Sustain. Energy Technol. Assess..

[5] Kulkarni S.J., Kaware J.P. (2013). Review on research for removal of phenol from wastewater. Int. J. Sci. Res. Publ..

[6] Feng Q., Zhao L., Lin J.-M. (2009). Molecularly imprinted polymer as micro-solid phase extraction combined with high performance liquid chromatography to determine phenolic compounds in environmental water samples. Anal. Chim. Acta.

[7] Logan B.E., Hamelers B., Rozendal R., Schröder U., Keller J., Freguia S., Aelterman P., Verstraete W., Rabaey K. (2006). Microbial fuel cells: Methodology and technology. Environ. Sci. Technol..

[8] Logan B.E. (2009). Exoelectrogenic bacteria that power microbial fuel cells. Nat. Rev. Microbiol..

[9] Li H., Tian Y., Zuo W., Zhang J., Pan X., Li L., Su X. (2016). Electricity generation from food wastes and characteristics of organic matters in microbial fuel cell. Bioresour. Technol..

[10] Logan B.E., Regan J.M. (2006). Microbial fuel cells—Challenges and applications. Environ. Sci. Technol..

[11] Hossain A., Masud N., Roy S., Ali M. (2022). Investigation of voltage storage capacity for the variation of electrode materials in microbial fuel cells with experimentation and mathematical modelling. Int. J. Water Resourc. Environ. Eng..

[12] Idris M.O., Ahmad A., Daud N.N.M. (2022). Removal of Toxic Metal Ions from Wastewater Through Microbial Fuel Cells. Microbial Fuel Cells for Environmental Remediation.

[13] Hossain A.M.A., Masud N., Yasin M.S., Ali M. (2020). Analysis of the performance of microbial fuel cell as a potential energy storage device. Proc. Int. Exch. Innov. Conf. Eng. Sci..

[14] Jia J., Tang Y., Liu B., Wu D., Ren N., Xing D. (2013). Electricity generation from food wastes and microbial community structure in microbial fuel cells. Bioresour. Technol..

[15] Asefi B., Li S.-L., Moreno H.A., Sanchez-Torres V., Hu A., Li J., Yu C.-P. (2019). Characterization of electricity production and microbial community of food waste-fed microbial fuel cells. Process. Saf. Environ. Prot..

[16] Yaqoob A.A., Mohamad Ibrahim M.N., Umar K., Bhawani S.A., Khan A., Asiri A.M., Khan M.R., Azam M., AlAmmari A.M. (2021). Cellulose Derived Graphene/Polyaniline Nanocomposite Anode for Energy Generation and Bioremediation of Toxic Metals via Benthic Microbial Fuel Cells. Polymers.

[17] Masud N., Hossain A.-M.A., Moresalein M.J., Ali M. (2021). Performance Evaluation of Microbial Fuel Cell with Food Waste Solution as a Potential Energy Storage Medium. Proc. Int. Exch. Innov. Conf. Eng. Sci..

[18] Goud R.K., Babu P.S., Mohan S.V. (2011). Canteen based composite food waste as potential anodic fuel for bioelectricity generation in single chambered microbial fuel cell (MFC): Bio-electrochemical evaluation under increasing substrate loading condition. Int. J. Hydrog. Energy.

[19] Moharir P.V., Tembhurkar A.R. (2018). Effect of recirculation on bioelectricity generation using microbial fuel cell with food waste leachate as substrate. Int. J. Hydrog. Energy.

[20] Rikame S.S., Mungray A.A., Mungray A.K. (2012). Electricity generation from acidogenic food waste leachate using dual chamber mediator less microbial fuel cell. Int. Biodeterior. Biodegrad..

[21] Asim A.Y., Mohamad N., Khalid U., Tabassum P., Akil A., Lokhat D., Siti H. (2021). A glimpse into the microbial fuel cells for wastewater treatment with energy generation. Desalination Water Treat..

[22] Azhari N.W. (2019). The Performance of Takakura Composting Using Food Waste from Makanan Ringan Mas Industry.

[23] Goud R.K., Mohan S.V. (2011). Pre-fermentation of waste as a strategy to enhance the performance of single chambered microbial fuel cell (MFC). Int. J. Hydrog. Energy.

[24] Daud N.N.M., Ahmad A., Yaqoob A.A., Ibrahim M.N.M. (2021). Application of rotten rice as a substrate for bacterial species to generate energy and the removal of toxic metals from wastewater through microbial fuel cells. Environ. Sci. Pollut. Res..

[25] Hassan S.H., Abd el Nasser A.Z., Kassim R.M. (2019). Electricity generation from sugarcane molasses using microbial fuel cell technologies. Energy.

[26] Simeon M.I., Asoiro F.U., Aliyu M., Raji O.A., Freitag R. (2020). Polarization and power density trends of a soil-based microbial fuel cell treated with human urine. Int. J. Energy Res..

[27] Rabaey K., Verstraete W. (2005). Microbial fuel cells: Novel biotechnology for energy generation. Trends Biotechnol..

[28] Prakash O., Mungray A., Kailasa S.K., Chongdar S., Mungray A.K. (2018). Comparison of different electrode materials and modification for power enhancement in benthic microbial fuel cells (BMFCs). Process. Saf. Environ. Prot..

[29] Abbas S.Z., Rafatullah M., Ismail N., Nastro R.A. (2017). Enhanced bioremediation of toxic metals and harvesting electricity through sediment microbial fuel cell. Int. J. Energy Res..

[30] Abbas S.Z., Rafatullah M., Ismail N., Shakoori F.R. (2018). Electrochemistry and microbiology of microbial fuel cells treating marine sediments polluted with heavy metals. RSC Adv..

[31] Ahmad A., Alshammari M.B. (2022). Basic principles and working mechanisms of microbial fuel cells. Microbial Fuel Cells: Emerging Trends in Electrochemical Applications.

[32] Rojas-Flores S., Benites S.M., La Cruz-Noriega D., Cabanillas-Chirinos L., Valdiviezo-Dominguez F., Quezada Álvarez M.A., Vega-Ybañez V., Angelats-Silva L. (2021). Bioelectricity Production from Blueberry Waste. Processes.

[33] Fricke K., Harnisch F., Schröder U. (2008). On the use of cyclic voltammetry for the study of anodic electron transfer in microbial fuel cells. Energy Environ. Sci..

[34] López Zavala M.Á., Gonzalez Pena O.I., Cabral Ruelas H., Delgado Mena C., Guizani M. (2019). Use of cyclic voltammetry to describe the electrochemical behavior of a dual-chamber microbial fuel cell. Energies.

[35] Torres C.I., Marcus A.K., Lee H.-S., Parameswaran P., Krajmalnik-Brown R., Rittmann B.E. (2010). A kinetic perspective on extracellular electron transfer by anode-respiring bacteria. FEMS Microbiol. Rev..

[36] Wen H., Zhu H., Xu Y., Yan B., Shutes B., Bañuelos G., Wang X. (2021). Removal of sulfamethoxazole and tetracycline in constructed wetlands integrated with microbial fuel cells influenced by influent and operational conditions. Environ. Pollut..

[37] Yaqoob A.A., Ibrahim M.N.M., Yaakop A.S., Ahmad A. (2021). Application of microbial fuel cells energized by oil palm trunk sap (OPTS) to remove the toxic metal from synthetic wastewater with generation of electricity. Appl. Nanosci..

[38] Rodríguez-Couto S., Ahmad A. (2021). Preparation, characterization, and application of modified carbonized lignin as an anode for sustainable microbial fuel cell. Process. Saf. Environ. Prot..

[39] Inamdar A., Kim Y., Pawar S., Kim J., Im H., Kim H. (2011). Chemically grown, porous, nickel oxide thin-film for electrochemical supercapacitors. J. Power Sources.

[40] Dolatabadi M., Ahmadzadeh S. (2019). A rapid and efficient removal approach for degradation of metformin in pharmaceutical wastewater using electro-Fenton process; optimization by response surface methodology. Water Sci. Technol..

[41] Yaakop A.S., Rafatullah M. (2022). Utilization of biomass-derived electrodes: A journey toward the high performance of microbial fuel cells. Appl. Water Sci..

[42] Serrà A., Bhawani S.A., Ibrahim M.N.M., Khan A., Alorfi H.S., Asiri A.M., Hussein M.A., Khan I., Umar K. (2022). Utilizing biomass-based graphene oxide–polyaniline–ag electrodes in microbial fuel cells to boost energy generation and heavy metal removal. Polymers.

[43] Anwer A., Khan M., Khan N., Nizami A., Rehan M., Khan M. (2019). Development of novel MnO2 coated carbon felt cathode for microbial electroreduction of CO2 to biofuels. J. Environ. Manag..

[44] Bakar M.A.B.A., Kim H.-C., Ahmad A., Alshammari M.B., Yaakop A.S. (2022). Oxidation of food waste as an organic substrate in a single chamber microbial fuel cell to remove the pollutant with energy generation. Sustain. Energy Technol. Assess..

[45] He Z., Mansfeld F. (2009). Exploring the use of electrochemical impedance spectroscopy (EIS) in microbial fuel cell studies. Energy Environ. Sci..

[46] Hung Y.-H., Liu T.-Y., Chen H.-Y. (2019). Renewable coffee waste-derived porous carbons as anode materials for high-performance sustainable microbial fuel cells. ACS Sustain. Chem. Eng..

[47] Idris M.O., Noh N.A.M. (2023). Sustainable microbial fuel cell functionalized with a bio-waste: A feasible route to formaldehyde bioremediation along with bioelectricity generation. Chem. Eng. J..

[48] Nevin K.P., Kim B.-C., Glaven R.H., Johnson J.P., Woodard T.L., Methé B.A., DiDonato R.J., Covalla S.F., Franks A.E., Liu A. (2009). Anode biofilm transcriptomics reveals outer surface components essential for high density current production in Geobacter sulfurreducens fuel cells. PLoS ONE.

[49] Dearden J., Forbes W. (1959). Light absorption studies: Part XIV. The ultraviolet absorption spectra of phenols. Can. J. Chem..

[50] Kowalski R., Kowalska G. (2005). Phenolic acid contents in fruits of aubergine (*Solanum melongena* L.). Pol. J. Food Nutr. Sci..

[51] Craft B.D., Kerrihard A.L., Amarowicz R., Pegg R.B. (2012). Phenol-based antioxidants and the in vitro methods used for their assessment. Compr. Rev. Food Sci. Food Saf..

[52] Umar M.F., Rafatullah M., Abbas S.Z., Ibrahim M.N.M., Ismail N. (2021). Bioelectricity production and xylene biodegradation through double chamber benthic microbial fuel cells fed with sugarcane waste as a substrate. J. Hazard. Mater..

[53] Serrà A., Yaqoob A.A., Ibrahim M.N.M., Yaakop A.S. (2021). Self-assembled oil palm biomass-derived modified graphene oxide anode: An efficient medium for energy transportation and bioremediating Cd (II) via microbial fuel cells. Arab. J. Chem..

[54] Reimers C.E., Li C., Graw M.F., Schrader P.S., Wolf M. (2017). The identification of cable bacteria attached to the anode of a benthic microbial fuel cell: Evidence of long distance extracellular electron transport to electrodes. Front. Microbiol..

[55] Lovley D.R. (2017). Electrically conductive pili: Biological function and potential applications in electronics. Curr. Opin. Electrochem..

[56] Guang L., Koomson D.A., Jingyu H., Ewusi-Mensah D., Miwornunyuie N. (2020). Performance of exoelectrogenic bacteria used in microbial desalination cell technology. Int. J. Environ. Res. Public Health.

[57] Aleid G.M., Alshammari A.S., Alomari A.D., Almukhlifi H.A., Ahmad A., Yaqoob A.A. (2023). Dual role of sugarcane waste in benthic microbial fuel to produce energy with degradation of metals and chemical oxygen demand. Processes.

[58] Nimje V.R., Chen C.-Y., Chen C.-C., Jean J.-S., Reddy A.S., Fan C.-W., Pan K.-Y., Liu H.-T., Chen J.-L. (2009). Stable and high energy generation by a strain of Bacillus subtilis in a microbial fuel cell. J. Power Sources.

[59] Torlaema T.A.M., Ibrahim M.N.M., Ahmad A., Guerrero-Barajas C., Alshammari M.B., Oh S.-E., Hussain F. (2022). Degradation of Hydroquinone Coupled with Energy Generation through Microbial Fuel Cells Energized by Organic Waste. Processes.

[60] Djukić-Vuković A., Meglič S.H., Flisar K., Mojović L., Miklavčič D. (2021). Pulsed electric field treatment of Lacticaseibacillus rhamnosus and Lacticaseibacillus paracasei, bacteria with probiotic potential. LWT.

[61] Malik S., Kishore S., Dhasmana A., Kumari P., Mitra T., Chaudhary V., Kumari R., Bora J., Ranjan A., Minkina T. (2023). A Perspective Review on Microbial Fuel Cells in Treatment and Product Recovery from Wastewater. Water.

[62] He L., Du P., Chen Y., Lu H., Cheng X., Chang B., Wang Z. (2017). Advances in microbial fuel cells for wastewater treatment. Renew. Sustain. Energy Rev..

[63] Yaqoob A.A., Ibrahim M.N.M., Umar K. (2021). Biomass-derived composite anode electrode: Synthesis, characterizations, and application in microbial fuel cells (MFCs). J. Environ. Chem. Eng..

[64] Yaqoob A.A., Ibrahim M.N.M., Yaakop A.S. (2021). Application of oil palm lignocellulosic derived material as an efficient anode to boost the toxic metal remediation trend and energy generation through microbial fuel cells. J. Clean. Prod..

[65] Idris M.O., Guerrero–Barajas C., Kim H.-C. (2022). Scalability of biomass-derived graphene derivative materials as viable anode electrode for a commercialized microbial fuel cell: A systematic review. Chin. J. Chem. Eng..

[66] Ahmad A., Ibrahim M.N.M., Yaqoob A.A., Setapar S.H.M. (2022). Microbial Fuel Cells for Environmental Remediation.

[67] Fadzli F., Ibrahim M., Yaakop A. (2023). Benthic microbial fuel cells: A sustainable approach for metal remediation and electricity generation from sapodilla waste. Int. J. Environ. Sci. Technol..

[68] Al-Zaqri N., Yaakop A.S., Umar K. (2022). Potato waste as an effective source of electron generation and bioremediation of pollutant through benthic microbial fuel cell. Sustain. Energy Technol. Assess..

[69] Guerrero–Barajas C., Ibrahim M.N.M., Umar K., Yaakop A.S. (2022). Local fruit wastes driven benthic microbial fuel cell: A sustainable approach to toxic metal removal and bioelectricity generation. Environ. Sci. Pollut. Res..

[70] Fadzli F.S., Rashid M., Yaqoob A.A., Ibrahim M.N.M. (2021). Electricity generation and heavy metal remediation by utilizing yam (Dioscorea alata) waste in benthic microbial fuel cells (BMFCs). Biochem. Eng. J..

[71] Khatoon A., Mohd Setapar S.H., Parveen T. (2020). Outlook on the role of microbial fuel cells in remediation of environmental pollutants with electricity generation. Catalysts.

[72] Ibrahim M.N.M., Yaqoob A.A., Ahmad A. (2022). Microbial Fuel Cells: Emerging Trends in Electrochemical Applications.

