# Supplementary material for: Bioenergy Generation and Phenol Degradation through Microbial Fuel Cells Energized by Domestic Organic Waste
Source: Molecules. 2023 May 25;28(11):4349. doi: 10.3390/molecules28114349 (PMC10254430; doi:10.3390/molecules28114349)
Supplement: Supplementary file 1 [file molecules-28-04349-s001.zip › molecules-2418486-supplementary.pdf]

Supporting file

Bioenergy Generation and Phenol Degradation through Microbial Fuel Cells Energized by Domestic Organic Waste

Asim Ali Yaqoob[1], Nabil Al-Zaqri[2], Muhammad Alamzeb[3], Fida Hussain [4], Sang-Eun Oh[5], Khalid Umar[1*]

[1] School of Chemical Sciences, Universiti Sains Malaysia, 11800 Minden, Penang, Malaysia

[2] Department of Chemistry, College of Science, King Saud University, P.O. Box 2455, Riyadh 11451, Saudi Arabia

[3] Department of Chemistry, University of Kotli, Kotli-11100, Azad Jammu & Kashmir, Pakistan.

[4] Research Institute for Advanced Industrial Technology, College of Science and Technology, Korea University, Sejong 30019, Republic of Korea

[5] Department of Biological Environment, Kangwon National University, Chuncheon-si, South Korea

*Corresponding author: khalidumar4@gmail.com

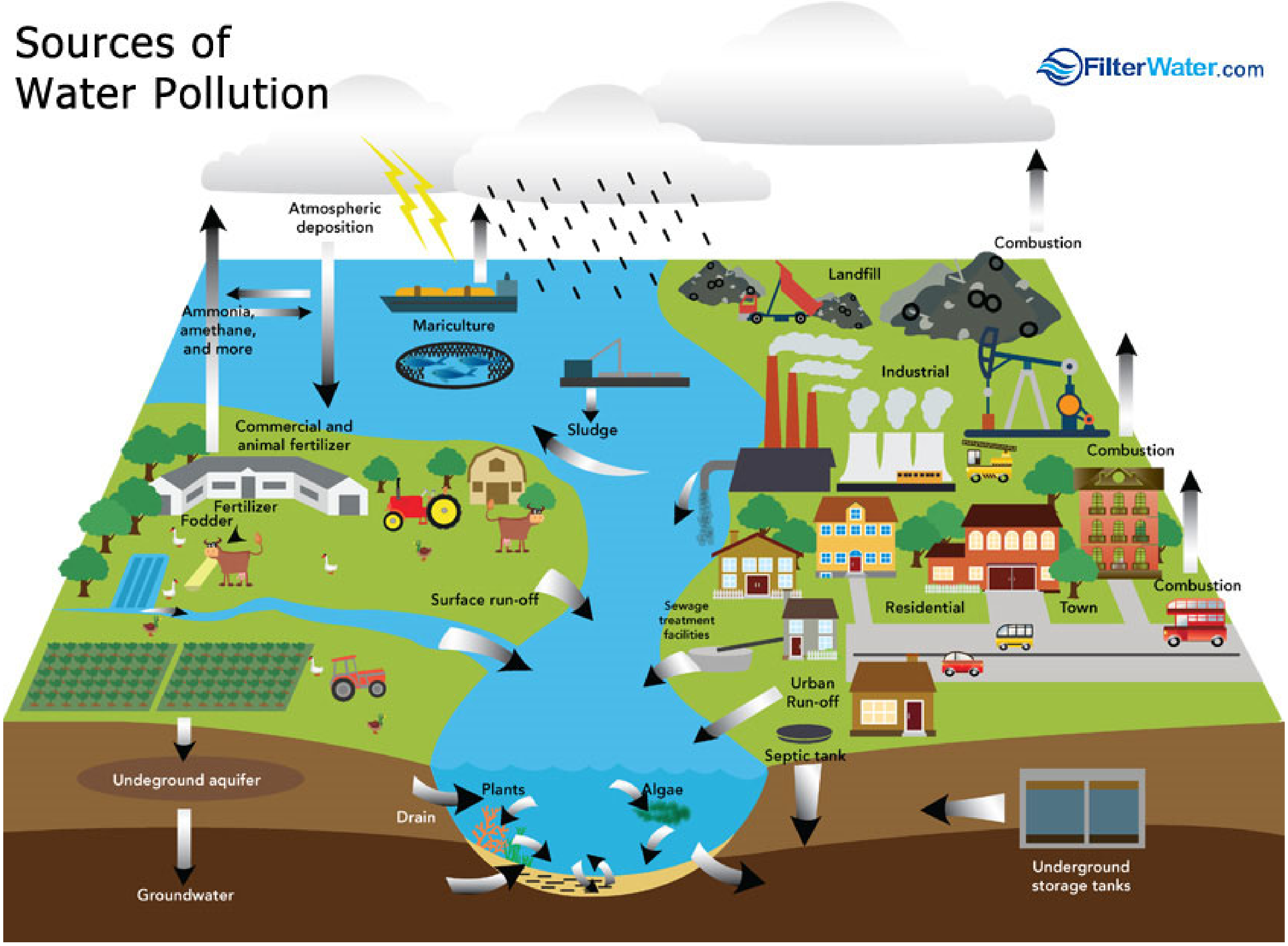

Figure S1: Different sources of water pollution (Adapted from: https://www.filterwater.com/t-articles.water-pollution.aspx).

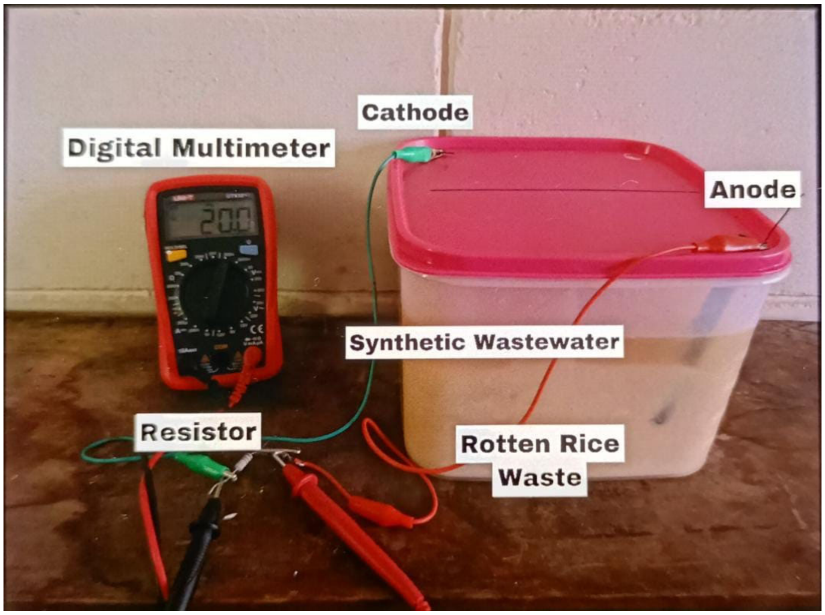

Figure S2: MFCs Set-up for phenol degradation.

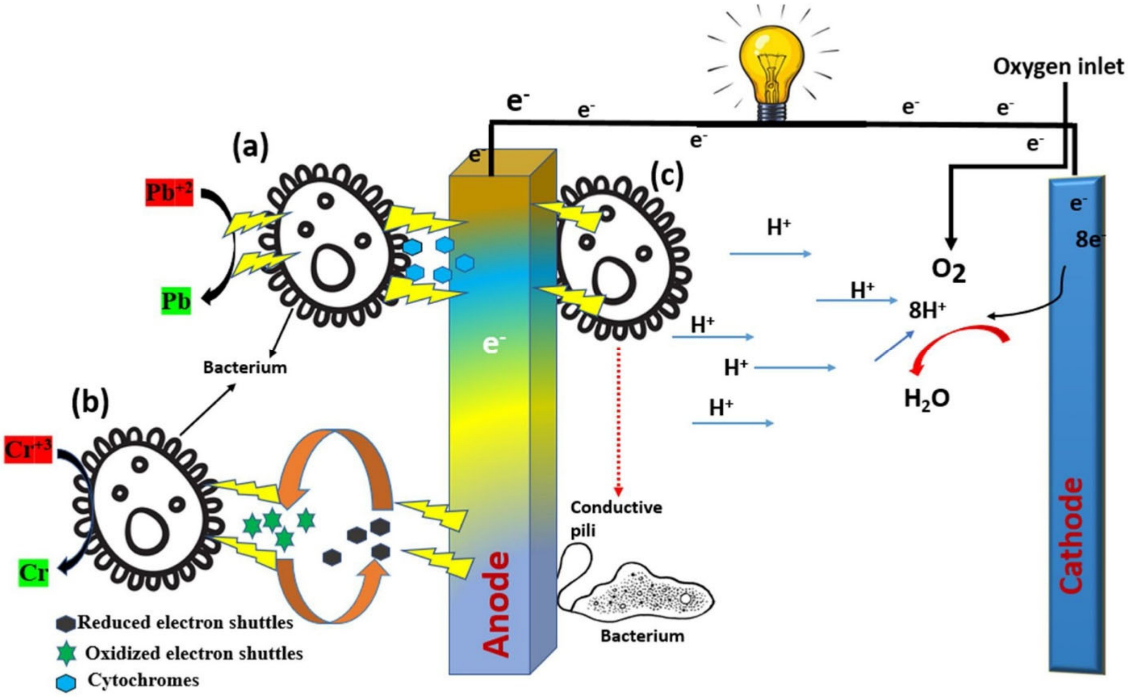

Figure S3: Different mechanisms of the electron transfer from bacterial species to anode electrode in MFCs (Adapted from reference [1] with Elsevier permission).

NOTE: In the literature, which we have cited here, the processes of electron transport from bacterial species to the anode electrode in MFCs are well explained [2-5].

References:

1. Fadzli, F.S.; Rashid, M.; Yaqoob, A.A.; Ibrahim, M.N.M. Electricity generation and heavy metal remediation by utilizing yam (Dioscorea alata) waste in benthic microbial fuel cells (BMFCs). *Biochemical Engineering Journal* **2021**, *172*, 108067.
2. Daud, N.N.M.; Ahmad, A.; Yaqoob, A.A.; Ibrahim, M.N.M. Application of rotten rice as a substrate for bacterial species to generate energy and the removal of toxic metals from wastewater through microbial fuel cells. *Environmental Science and Pollution Research* **2021**, *28*, 62816-62827.
3. Khatoon, A.; Mohd Setapar, S.H.; Parveen, T. Outlook on the role of microbial fuel cells in remediation of environmental pollutants with electricity generation. *Catalysts* **2020**, *10*, 819.
4. Ibrahim, M.N.M.; Yaqoob, A.A.; Ahmad, A. *Microbial Fuel Cells: Emerging trends in electrochemical applications*; IOP Publishing: 2022.
5. Ahmad, A.; Ibrahim, M.N.M.; Yaqoob, A.A.; Setapar, S.H.M. *Microbial Fuel Cells for Environmental Remediation*; Springer Nature: 2022.
